# Biography of a Fool

**Published:** 1905-02

**Authors:** 


### BIOGRAPHY OF A FOOL.

He didn't have time to chew  
The food that he had to eat,  
But he washed it into his throat  
As if time were a thing to beat.  
At breakfast and lunch and dinner  
'Twas a bite and a gulp and go—  
Oh, the crowd is so terribly eager,  
And a man has to hurry so!

A bite and a gulp and away  
To the books and the ticker! A bite  
And a drink and a smoke and a seat  
At a card-table half of the night;  
A pressure, a click and a pallor,  
A cloth-covered box and a song;  
A weary old fellow at forty,  
Who is deaf to the noise of the throng.

—*Chicago Times-Herald.*

